# Pharmacists’ Perspectives on the Use of My Health Record

**DOI:** 10.3390/pharmacy8040190

**Published:** 2020-10-14

**Authors:** Sam Kosari, Kwang Choon Yee, Stephanie Mulhall, Jackson Thomas, Shane L. Jackson, Gregory M. Peterson, Ayla Rudgley, Iain Walker, Mark Naunton

**Affiliations:** 1 Discipline of Pharmacy, Faculty of Health, University of Canberra, Bruce 2617, Australia; Kwang.Yee@canberra.edu.au (K.C.Y.); stephanie.mulhall@anu.edu.au (S.M.); jackson.thomas@canberra.edu.au (J.T.); g.peterson@utas.edu.au (G.M.P.); aylarudgley@gmail.com (A.R.); mark.naunton@canberra.edu.au (M.N.); 2 Health Research Institute, Faculty of Health, University of Canberra, Bruce 2617, Australia; 3 Research School of Psychology, Australian National University, Canberra 2600, Australia; Iain.Walker@anu.edu.au; 4 Discipline of Pharmacy, School of Medicine, University of Tasmania, Hobart 7005, Australia; shane.jackson@utas.edu.au

**Keywords:** my health record, pharmacist, pharmacists’ perspective on digital health, electronic health records, community pharmacy

## Abstract

(1) Background: My Health Record (MHR) is a relatively new nationwide Australian digital health record system accessible by patients and a range of healthcare professionals. Pharmacists will be key contributors and users of the MHR system, yet little is known about the perceived barriers and benefits of use. (2) Objective: To explore pharmacists’ perspectives related to potential benefits and barriers associated with use of MHR. (3) Methods: An online survey was developed and face-validated. The survey was advertised to Australian pharmacists on pharmacy professional bodies’ websites. This was a cross-sectional study using an anonymous questionnaire. Descriptive statistics were used to describe the distribution of the data. Chi-square, Kendall’s tau coefficient (tau-c) and Kruskal–Wallis tests were used to examine the relationships where appropriate. (4) Results: A total of 63 pharmacists completed the survey. The majority of respondents worked in a metropolitan area (74%), and the most common workplace setting was community pharmacy (65%). Perceived benefits identified by responders include that the use of MHR would help with continuity of care (90%), and that it would improve the safety (71%) and quality (75%) of care they provided. Importantly, more than half of pharmacists surveyed agreed that MHR could reduce medication errors during dispensing (57%) and could improve professional relationships with patients (57%) and general practitioners (59%). Potential barriers identified by pharmacists included patients’ concerns about privacy (81%), pharmacists’ own concern about privacy (46%), lack of training, access to and confidence in using the system. Sixty six percent of respondents had concerns about the accuracy of information contained within MHR, particularly among hospital and general practice pharmacists (*p* = 0.016) and almost half (44%) had concerns about the security of information in the system, mainly pharmacists working at general practice and providing medication review services (*p* = 0.007). Overall satisfaction with MHR varied, with 48% satisfied, 33% neither satisfied nor dissatisfied, and 19% dissatisfied, with a higher satisfaction rate among younger pharmacists (*p* = 0.032). (5) Conclusions: Pharmacists considered that the MHR offered key potential benefits, notably improving the safety and quality of care provided. To optimize the use of MHR, there is a need to improve privacy and data security measures, and to ensure adequate provision of user support and education surrounding the ability to integrate use of MHR with existing workflows and software.

## 1. Introduction

Advancements in information technology have numerous potential applications in healthcare, including the adoption of electronic health records (EHRs) in several countries [1,2,3]. According to the World Health Organization, more than 50% of upper-middle and high-income countries (e.g., countries in North America and Europe) have adopted national EHR systems [4].The Australian Government initially introduced shared personally-controlled EHRs in 2012; these were relaunched and renamed in 2016 as My Health Record (MHR). MHR aims to improve the safety and efficiency of healthcare by reducing the potential for medical errors, facilitating collaboration among health professionals and encouraging patient participation and engagement through individual access to a summary of their clinical records [5,6,7]. Australian MHR contains a summary of an individual’s health information such as list of medical conditions, medications, pathology tests or scans results, hospital discharge notes, immunization records and organ donation decisions. Patients can also update their information. Health professionals can upload relevant medical documents such as shared health summary, medical doctors’ consultation detail, pharmacists’ medication review notes and care plans [8].

The use of MHR in pharmacy is growing, with 83% of Australian pharmacies [9] (approximately 5700) [10] reportedly registered with access to the system, as of April 2019 [9]. The number of pharmacies that actually use MHR routinely has not been reported to date; however, pharmacists’ use of MHR is expected to improve over time. This is likely to improve pharmacists’ access to patient information, medication history, known allergies, medical conditions and hospital discharge summaries, in a consolidated environment. Access to an integrated patient summary (shared health summary) through MHR has the potential to improve quality of care, reduce medication-related problems and improve interprofessional collaboration [9].

Achieving and utilising the full potential of MHR will require consumers and healthcare providers to actively engage with the system; thus, greater understanding of the perspectives of user groups and profession-specific barriers and benefits to the use of MHR is important. Previous studies have sought to examine the attitudes of healthcare consumers [11,12,13] and a range of healthcare providers [13,14,15,16] towards the use of personally-controlled EHRs, yet the unique perspectives of pharmacists as key users of MHR have not been previously investigated. The expanded role of pharmacists in healthcare delivery requires access to comprehensive patient records. Pharmacists in Australia conduct a variety of clinical activities based on their practice settings that are commonly characterized as community, hospital, consultant, industrial or academic pharmacists [17,18]. Pharmacists have frequent contact with patients in community pharmacy settings and could play an important role in providing quality information about medication therapies on EHRs [19]. This study aimed to gather the pharmacists’ perspectives about the benefits and barriers of using MHR in their professional role.

## 2. Methods

A survey questionnaire (Supplementary Materials) targeting Australian registered pharmacists was developed that consisted of 5 domains: (i) characteristics of respondents and their frequency of use of MHR; perceived (ii) benefits, (iii) barriers and (iv) concerns about the use of MHR; and (v) overall satisfaction. Respondents were presented with a series of statements and asked to indicate either strength of agreement or disagreement (ranging from ‘strongly agree’ to ‘strongly disagree’) or the likelihood that the barrier would apply to them (ranging from ‘extremely likely’ to ‘extremely unlikely’) using a 5-point Likert scale. A free-text box was provided for respondents to describe other barriers to the use of MHR and characteristics of MHR that they believed required change or improvement. The survey was face-validated by collaborators and registered pharmacists.

The survey was simultaneously promoted through the following pharmacy-related newsletters and websites: the Pharmacy Guild of Australia (website only), Pharmaceutical Society of Australia (website only), Australian Association of Consultant Pharmacy (newsletter only), and the Australian Journal of Pharmacy (newsletter and website). The survey was hosted/available on QUALTRICS and was accessible for respondents between September 2018 and March 2019. A reminder was also distributed through pharmacy professional bodies’ websites during this period.

Descriptive statistics were used to describe the distribution of the data. Categories of agreement and disagreement (strongly, somewhat) and likelihood (extremely, somewhat) were collapsed to improve interpretation in the text, while complete data is presented in table form. Analytical statistics were performed when appropriate. Chi-square and Kendall’s tau coefficient (tau-c) tests were used to examine the relationship between the ordinal data; and Kruskal–Wallis test was used to examine the relationship between a nominal data vs ordinal data. Statistical analyses were performed using SPSS version 25 (IBM, Armonk, NY, USA).

All participants provided their informed consent electronically before they participated in the study. The study was conducted in accordance with the Declaration of Helsinki, and the protocol was approved by the University of Canberra Human Research Ethics Committee (approval number HREC 1510). This was an anonymous voluntary study and no identifiable information was collected.

## 3. Results

A total of 63 respondents from all Australian states and territories completed the online survey with valid responses. The survey was conducted at an early stage of re-launch of MHR; there were approximately 30,000 registered pharmacists in Australia in 2018 [20]; however, it is unknown how many pharmacists actively used MHR or read the survey advertisement on pharmacy professional bodies.

Demographic characteristics are presented in Table 1. Most respondents (84%) were aged between 25–64 years and 57% were female—this is consistent with the gender distribution of pharmacists registered in Australia [19]. The majority of respondents practiced in a metropolitan rather than rural area (75% vs 25%), and the most commonly reported primary patient care role was community pharmacy (65%), followed by conducting Home Medicines Reviews (HMR) or Residential Medication Management Reviews (RMMR) (17%), hospital pharmacy (14%) and general practice clinics (3%).

Nearly two-thirds of respondents had current access to MHR (65%); others reported they did not have access (24%) or were unsure if they had access (11%). Of those with access to MHR, 41% had accessed MHR between 1–5 times during the past month, while 17% had accessed MHR 6–10 times. Most respondents (63%) indicated that they were likely to use MHR within the next 12 months.

### 3.1. Benefits

Responses to statements related to potential benefits of MHR are displayed in Table 2. The majority of respondents agreed (either somewhat or strongly) that use of MHR would help with continuity of care (90%) and improve the safety (71%) and quality (75%) of the care they provided. Over half of the respondents (57%) agreed that use of MHR has the potential to reduce medication errors during the dispensing process. Most agreed that use of MHR could improve their professional relationship with patients (57%) and general practitioners (59%). We observed that respondents who used MyHR more frequently in the past month, were more likely to agree that MHR ‘will help with continuity of care’ (Kendall’s tau-c *p* = 0.001). Other demographic factors did not appear to be significantly influence the perceived benefits.

### 3.2. Barriers

Participant responses to statements related to potential barriers to MHR use are displayed in Table 3. Eighty-one percent of responding pharmacists reported that patients’ concerns about privacy or confidentiality were likely (either extremely likely or somewhat likely) to act as a barrier to the use of MHR. Pharmacists’ own concerns about privacy and confidentiality were also considered likely to act as a barrier by 46% of respondents. Some respondents reported a lack of IT support (30%), training to set up and access the system (41%), and training to enable users to confidently use the system (41%) as likely barriers. A proportion of respondents (21%) identified use of MHR as being likely to interfere with existing dispensing processes, while over 40% reported this as unlikely (somewhat or extremely unlikely) to act as a barrier. Almost half of respondents (48%) expressed that it was ‘neither likely nor unlikely’ that their workplace would not promote use of the system.

### 3.3. Concerns

A review of the statements with possible concerns and participant responses are displayed in Table 4. Two-thirds of respondents had concerns about the accuracy of information contained within MHR and almost half (44%) had concerns about the security of information in the system. Thirty-eight percent agreed that they were concerned about the system not being user-friendly to navigate and find information, while 30% agreed that MHR would slow down the dispensing process. Respondents whose primary practice were hospital or GP clinic expressed greater concerns about the accuracy of MHR data (Kruskal–Wallis test, *p* = 0.016); and those pharmacists whose primary roles were HMR/RMMR providers or in GP clinics had greater concerns about the security of MHR data (Kruskal–Wallis test, *p* = 0.007). Additionally, respondents that had greater concern about the accuracy of MHR information were less likely to use the system (Kendall’s tau-c, *p* = 0.009).

### 3.4. Overall Satisfaction

Overall satisfaction with MHR varied, with most being satisfied (extremely or somewhat; 48%), a lesser proportion reported they were neither satisfied or dissatisfied (33%), and the remaining respondents reported dissatisfaction (extremely or somewhat; 19%). Younger pharmacists (age < 45) were more satisfied with MHR than older pharmacists (age > 45) (Kruskal–Wallis test, *p* = 0.032).

### 3.5. Other Comments

Respondents were asked about the characteristics of MHR that required improvement, using a free-text response. The following themes emerged: privacy and security of patient data; improving the user interface and ease of use; training and the provision of appropriate, timely support for system users; and further improvements in integration with dispensing software.

## 4. Discussion

This study explored the perspectives of a sample of pharmacists regarding the use of MHR at an early stage of widespread re-launch of MHR in Australia. Findings indicated a reasonably high level of awareness and access to MHR among the pharmacists surveyed. Most respondents believed that MHR is likely to be beneficial in many aspects such as reducing errors, improving patient care, enhancing collaboration with health professionals and improving patient satisfaction. Younger pharmacists had a higher satisfaction rate with MHR overall, which may be due to greater familiarity with electronic communication. However, pharmacists mainly from hospital pharmacies or general practice clinics, expressed concerns about accuracy and completeness of the information, which is essential in achieving the full benefit of MHR. We believe this might be due to the amount of information available in MHR at present. While this information is more comprehensive than records kept at community pharmacies, it is less comprehensive than records kept at hospital or general practice clinics.

More than half of the pharmacists surveyed agreed that use of MHR could potentially reduce medication errors when dispensing and might improve the safety of the care provided. Given that one of the primary roles of pharmacists is to facilitate the safe delivery of medication and minimise medication misadventure [21], the use of MHR may offer benefits that are closely aligned with pharmacists’ core professional activities. Greater access to an integrated patient record has the potential to improve safety outcomes, particularly during care transitions and for patients with increased risk of medication-related problems, such as older people [22]. Previous evidence has demonstrated that use of EHRs in the hospital setting can reduce medication-related problems [23]. In the community pharmacy setting, a lack of hospital discharge communication acts as a barrier to medication reconciliation [24], which can be reduced with use of EHRs [25]. Community pharmacies are accessible and highly utilised by health care consumers, with one large study finding that the first contact with a health care provider following hospital discharge was with a community pharmacist [26]. Yet, routine communication following hospital discharge is low, and community pharmacists have relied on patients relaying medication information. Medication-related problems that occur when patients transition between care settings may be reduced through improved interprofessional collaboration and the use of a shared system [7]. A 2018 study that examined the use of EHRs to facilitate a comprehensive medication review following hospital discharge, found a significant reduction in hospital readmission in patients who underwent the review following discharge compared to those who did not [27].

Rather than relying on patient recall alone, the use of patient-entered Health Summaries and pharmacist-entered information in a pharmacist shared medicines list on MHR may also contribute to a reduction in drug interactions between prescription and non-prescription medication. Greater access to relevant patient information creates opportunities for pharmacists to contribute to optimised treatment [22]. Digital information transfer such as through an EHR improves the speed and accuracy of information, and was considered the preferred mode of receiving information in a 2018 study of pharmacists [24].

The appropriate management of patient confidentiality, privacy and data security is essential to the successful implementation of EHRs [28]. Given the sensitive nature of personal health data and its potential for misuse, it is unsurprising that pharmacists in this study identified these issues as potential barriers to the widespread adoption of MHR. While almost half of respondents reported their own concerns related to patient privacy and confidentiality as a likely barrier to use, even more (81%) considered patient-related concerns about privacy and confidentiality a barrier. The security of patient health records and trust in the management of health-related personal data remain key issues to the adoption of EHRs, with previous studies finding that concerns about privacy, confidentiality and data security were common among a range of users including general practitioners, nurses, and allied health practitioners [14,15,29]. Pharmacists’ perceptions of patient-related concerns limiting the use of MHR are not unfounded, with patient concerns about privacy associated with use of personal health records previously identified [12,30]. Yet, the concept of personally-controlled EHRs has been well received by some consumers who see the potential for improved communication with providers, better quality healthcare and improved ability to self-manage their health [10,12,15]. With more than 90% of Australians registered for an MHR (following the voluntary opt-out process), as of early 2019 [31], it is likely that perceptions about patient-related concerns will change over time.

In a systematic review of user perspectives of factors influencing implementation of EHR, technical aspects were identified as one of the most common barriers [32]. Similarly, ease of use and technical concerns have been identified as barriers to MHR use among general practitioners, nurses and other allied health professionals [14], while a perceived increase in workload and time-related demands were barriers to pharmacists [33] and other health care providers [14]. Respondents in the present study indicated that a lack of training during setup, along with a lack of training to ensure users could confidently use MHR, was likely to act as a barrier to the use of MHR. Pharmacists also expressed concerns that the use of MHR is likely to interfere with the existing dispensing processes and may slow down the workflow. This is particularly important for community pharmacies, operating as small businesses. Perceived ease of use has been identified as a strong predictor of intention to use EHRs [34] and can facilitate implementation [32,35]. Findings revealed that improving the integration between existing dispensing software and MHR was important for pharmacists in this study.

Two-thirds of pharmacists surveyed in this study agreed that the accuracy of the information contained within MHR was a concern. This could be potentially related to unfamiliarity with the MHR system, limitations of incomplete records and where information had been omitted or was concealed from view. From a pharmacist perspective, especially in community pharmacy, extra information available within the MHR may shed further light on the medical conditions and other relevant information about a patient. Similar concerns were reported in a qualitative study of Australian pharmacists before the relaunch of MHR [33]. The Pharmaceutical Society of Australia briefly addresses issues of completeness in their My Health Record Guidelines for Pharmacists and states that MHR “cannot be assumed to be a complete record” [36]. Personal control over what is contained in an individual’s MHR and what is subsequently shared with health care providers is a key feature of MHR. While this level of patient control may have disadvantages and the absence of all information may influence the healthcare provider’s decision making, it offers active users a level of protection, which is important given the range of sensitive information a record may contain and the varied information needs of the healthcare providers. This might be minimised if there is an indicator showing where information is removed.

In order to optimize MHR and further facilitate the use of MHR in pharmacies, stakeholders’ concerns should be considered. To address the concerns about accuracy of data and user-friendliness, we recommend MHR to adopt standardization, integration, and synchronization of data with existing systems including pharmacy dispensing systems and hospital digital health records [37,38,39]. We also recommend programs to encourage pharmacists to contribute towards ongoing improvement of MHR and improve pharmacist partnership in utilizing MHR, such as updating patient-level information and incorporating interventions or recommendations in MHR [38,40,41].

## 5. Limitations

The study reflects on findings from a relatively small sample (*n* = 63) out of a total of 30,000 registered pharmacists in Australia. It was unknown how many pharmacists actively used MHR or whether the survey was accessible to all Australian pharmacists. Additionally, respondents were mainly from the metropolitan areas. Hence, overall results may not be generalizable. Since the MHR had been recently relaunched, at the time of the survey, some respondents had not yet accessed MHR in their workplace and user perspectives may change with greater exposure to the system.

## 6. Conclusions

This study explored the perspectives of pharmacists regarding the use of MHR at an early stage of widespread re-launch of MHR in Australia. Most pharmacists identified benefits of MHR that were central to their role, including improved medication safety, continuity of care, and quality of care. The results suggest a need to continue to manage concerns related to privacy and data security, and to ensure adequate provision of user support and education surrounding the ability to integrate use of MHR with existing workflows and software. Further research is needed in the future with larger sample size to determine how the use of MHR by pharmacists may affect clinical outcomes and whether the perspectives of pharmacists change over time when the use of MHR is further expanded.

## Figures and Tables

**Table 1:** Demographic characteristics (*n* = 63).

Characteristic	*n* (%)
Age in years, *n* (%)	
<25	4 (6)
25–34	17 (27)
35–44	16 (25)
45–54	12 (19)
55–64	8 (13)
65+	3 (5)
Prefer not to answer	3 (5)
Gender *	
Male	26 (41)
Female	36 (57)
State of practice *	
ACT	10 (16)
NSW	22 (35)
NT	2 (3)
QLD	10 (16)
SA	1 (2)
TAS	3 (5)
WA	7 (11)
VIC	5 (8)
Area of practice	
Metropolitan	47 (75)
Rural	16 (25)

* Percentages reported are actual and do not equal 100% due to missing data/unanswered questions. ACT: Australian Capital Territory; NSW: New South Wales; NT: Northern Territory; QLD: Queensland; SA: South Australia; TAS: Tasmania; WA: Western Australia; VIC: Victoria.

**Table 2:** Perceived benefits of the use of My Health Record.

Statement	Response, *n* (%)				
	Strongly Agree	Somewhat Agree	Neither Agree Nor Disagree	Somewhat Disagree	Strongly Disagree
MHR will help with continuity of care	31 (49)	26 (41)	5 (8)	0 (0)	1 (2)
MHR reduces medication errors when dispensing	13 (21)	23 (36)	17 (27)	8 (13)	2 (3)
MHR improves the safety of care I provide	21 (33)	24 (38)	15 (24)	3 (5)	0 (0)
MHR improves the quality of patient care I provide	22 (35)	25 (40)	11 (17)	3 (5)	2 (3)
MHR improves my professional relationship with my patients	19 (30)	17 (27)	20 (32)	5 (8)	2 (3)
MHR improves my professional relationship with general practitioners	15 (24)	22 (35)	17 (27)	7 (11)	2 (3)

Abbreviations: MHR, My Health Record.

**Table 3:** Barriers to use of My Health Record.

Statement	Response, *n* (%)				
	Extremely Likely	Somewhat Likely	Neither Likely Nor Unlikely	Somewhat Unlilkely	Extremely Unlikely
I am concerned about privacy/confidentiality issues of MHR *	20 (32)	9 (14)	6 (9)	15 (24)	12 (19)
My patients are concerned about privacy/ confidentiality issues of MHR	25 (40)	26 (41)	9 (14)	3 (5)	0 (0)
My workplace does not promote the use of this system	3 (5)	11 (17)	30 (48)	8 (13)	11 (17)
I do not have the appropriate IT support	9 (14)	10 (16)	15 (24)	13 (21)	16 (25)
I have not received training to set up the access to the system	17 (27)	9 (14)	8 (13)	14 (22)	15 (24)
I have not received training to confidently use the system	15 (24)	11 (17)	9 (14)	11 (17)	17 (27)
It interferes with the existing dispensing processes	5 (8)	8 (13)	22 (35)	13 (21)	15 (24)
I do not believe the time spent provides a significant value gained	6 (9)	12 (19)	12 (19)	16 (25)	17 (27)
It is not within my position description in my current professional role	2(3)	6 (9)	14 (22)	12 (19)	29 (46)

* Percentages reported are actual and do not equal 100% due to missing data/unanswered questions. Abbreviations: MHR, My Health Record. Participant who identified that they were encountering issue such as lack of IT support and lack of training to setup MHR, were less likely to use MyHR (Kendall’s tau-c, *p* = 0.010 and *p* < 0.0001, respectively).

**Table 4:** Potential concerns to use of My Health Record.

Statement	Response, *n* (%) *				
	Strongly Agree	Somewhat Agree	Neither Agree Nor Disagree	Somewhat Disagree	Strongly Disagree
I have concerns about the security of information within the MHR system	18 (29)	10 (16)	10 (16)	15 (24)	10 (16)
I have concerns about accuracy of information within the MHR system	19 (30)	23 (36)	5 (8)	11 (17)	5 (8)
MHR slows me down when dispensing	12 (19)	7 (11)	24 (38)	10 (16)	10 (16)
It is not user-friendly to navigate MHR to find the information I want	10 (16)	14 (22)	28 (44)	8 (13)	3 (5)

* Percentages reported may not equal 100% due to rounding. Abbreviations: MHR, My Health Record.

## Supplementary Materials

The following are available online at https://www.mdpi.com/2226-4787/8/4/190/s1.

## References

[1] Gunter T.D., Terry N.P. (2005). The emergence of national electronic health record architectures in the United States and Australia: Models, costs, and questions. J. Med. Internet Res..

[2] Evans R.S. (2016). Electronic Health Records: Then, Now, and in the Future. Yearb. Med. Inform..

[3] Nøhr C., Parv L., Kink P., Cummings E., Almond H., Nørgaard J.R., Turner P. (2017). Nationwide citizen access to their health data: Analysing and comparing experiences in Denmark, Estonia and Australia. BMC Health Serv. Res..

[4] Whorld Health Organisation Global Health Observatory (GHO) Data: Electronic Health Records (Webpage). Updated 2020. https://www.who.int/gho/goe/electronic_health_records/en/.

[5] Australian Digital Health Agency, Australian Government Benefits of My Health Record for Healthcare Professionals (Webpage). Updated 2019. https://www.myhealthrecord.gov.au/for-healthcare-professionals/what-is-my-health-record/benefits-my-health-record-for-healthcare.

[6] Pearce C., Bainbridge M. (2014). A personally controlled electronic health record for Australia. J. Am. Med. Inform. Assoc..

[7] Jackson S., Peterson G. (2019). My Health Record: A community pharmacy perspective. Aust. Prescr..

[8] Australian Digital Health Agency, Australian Government My Health Record. What’s in a My Health Record? (Webpage). Updated: 2020. https://www.myhealthrecord.gov.au/for-you-your-family/whats-in-my-health-record#:~:text=My%20Health%20Record%20brings%20together,results%2C%20all%20in%20one%20place.

[9] Australian Digital Health Agency, Australian Government Media Release: Record Number of Sign Ups to My Health Record in Australian Pharmacies (Webpage). Updated 2019. https://www.myhealthrecord.gov.au/news-and-media/media-releases/sign-ups-my-health-record-pharmacies.

[10] The Pharmacy Guild of Australia Vital Facts on Community Pharmacy (Webpage). Updated May 2018. https://www.guild.org.au/__data/assets/pdf_file/0020/12908/Vital-facts-on-community-pharmacy.pdf.

[11] Hanna L., Gill S.D., Newstead L., Hawkins M., Osborne R.H. (2017). Patient perspectives on a personally controlled electronic health record used in regional Australia. Health Inf. Manag. J..

[12] Andrews L., Gajanayake R., Sahama T. (2014). The Australian general public’s perceptions of having a personally controlled electronic health record (PCEHR). Int. J. Med. Inform..

[13] Lehnbom E.C., Brien J.E., McLachlan A.J. (2014). Knowledge and attitudes regarding the personally controlled electronic health record: An Australian national survey. Intern. Med. J..

[14] Carroll J., Butler-Henderson K. (2017). MyHealthRecord in Australian Primary Health Care: An Attitudinal Evaluation Study. J. Med. Syst..

[15] Kariotis T.C., Harris K.M. (2019). Clinician perceptions of My Health Record in mental health care: Medication management and sharing mental health information. Aust. J. Prim. Health.

[16] Buss V.H., Shield A., Kosari S., Naunton M. (2018). The impact of clinical service provided by community pharmacies on the Australian healthcare system: A review of the literiture. J. Pharm. Policy Pract..

[17] Pharmaceutical Society of Australia Pharmacy as a Career (Webpage). Upated 2020. https://www.psa.org.au/career-and-support/pharmacy-as-a-career/.

[18] Nelson S.D., Poikonen J., Reese T., El Halta D., Weir C. (2017). The pharmacist and the HER. J. Am. Med. Inf. Assoc..

[19] Pharmacy Board of Australia, Australian Health Practioner Regulation Agency Statistic: Registration Data September 2018 (Webpage). Updated August 2020. https://www.pharmacyboard.gov.au/About/Statistics.aspx.

[20] Armani R., Mitchell L., Allen-Graham J., Heriot N., Kotsimbos T., Wilson J. (2016). Current patient and healthcare worker attitudes to eHealth and the personally controlled electronic health record in major hospitals. Intern. Med. J..

[21] Pharmaceutical Society of Australia (2016). National Competency Standards Framework for Pharmacists in Australia 2016.

[22] Bacci J.L., Berenbrok L.A. (2018). Innovative advances in connectivity and community pharmacist patient care services: Implications for patient safety. Pharmacother. J. Hum. Pharmacol. Drug Ther..

[23] Campanella P., Lovato E., Marone C., Fallacara L., Mancuso A., Ricciardi W., Specchia M.L. (2016). The impact of electronic health records on healthcare quality: A systematic review and meta-analysis. Eur. J. Public Health.

[24] Kennelty K.A., Chewning B., Wise M., Kind A., Roberts T., Kreling D. (2015). Barriers and facilitators of medication reconciliation processes for recently discharged patients from community pharmacists’ perspectives. Res. Soc. Adm. Pharm..

[25] Agrawal A. (2009). Medication errors: Prevention using information technology systems. Br. J. Clin. Pharmacol..

[26] Roughead E.E., Kalisch L.M., Ramsay E.N., Ryan P., Gilbert A.L. (2011). Continuity of care: When do patients visit community healthcare providers after leaving hospital?. Intern. Med. J..

[27] Fanizza F.A., Ruisinger J.F., Prohaska E.S., Melton B.L. (2018). Integrating a health information exchange into a community pharmacy transitions of care service. J. Am. Pharm. Assoc..

[28] Xu J., Gao X., Sorwar G., Croll P. (2013). Implementation of E-health Record Systems in Australia. Int. Technol. Manag. Rev..

[29] Lehnbom E.C., Douglas H.E., Makeham M.A.B. (2016). Positive beliefs and privacy concerns shape the future for the Personally Controlled Electronic Health Record. Intern. Med. J..

[30] Abdekhoda M., Dehnad A., Khezri H. (2019). The effect of confidentiality and privacy concerns on adoption of personal health record from patient’s perspective. Health Technol..

[31] Australian Government, Australian Digital Health Agency My Health Record Statistics (Webpage). Updated 2019. https://www.myhealthrecord.gov.au/statistics.

[32] McGinn C.A., Grenier S., Duplantie J., Shaw N., Sicotte C., Mathieu L., Leduc Y., Légaré F., Gagnon M.-P. (2011). Comparison of user groups’ perspectives of barriers and facilitators to implementing electronic health records: A systematic review. BMC Med..

[33] Mooranian A., Emmerton L., Hattingh L. (2013). The introduction of the national e-health record into A ustralian community pharmacy practice: Pharmacists’ perceptions. Int. J. Pharm. Pract..

[34] Gagnon M.-P., Ghandour E.K., Talla P.K., Simonyan D., Godin G., Labrecque M., Ouimet M., Rousseau M. (2014). Electronic health record acceptance by physicians: Testing an integrated theoretical model. J. Biomed. Inform..

[35] Gagnon M.-P., Desmartis M., Labrecque M., Car J., Pagliari C., Pluye P., Frémont P., Gagnon J., Tremblay N., Légaré F. (2012). Systematic Review of Factors Influencing the Adoption of Information and Communication Technologies by Healthcare Professionals. J. Med. Syst..

[36] Pharmaceutical Society of Australia (2017). My Health Record Guidelines for Pharmacists.

[37] Liaw S.T., Powell-Davies G., Pearce C., Britt H., McGlynn L., Harris M.F. (2016). Optimising the use of observational electronic health record data: Current issues, evolving opportunities, strategies and scope for collaboration. Aust. Phamily Physician.

[38] Weinfeld J.M., Mishori R. (2016). Toward electronic health record optimization: Learning from the user experience. J. Ambul. Care Manag..

[39] Australian Digital Health Agency, Australian Government Types of Digital Health Records (Webpage). Updated 2020. https://www.digitalhealth.gov.au/get-started-with-digital-health/digital-health-evidence-review/types-of-digital-health-records.

[40] Pandhi N., Yang W.L., Karp Z., Young A., Beasley J.W., Kraft S., Carayon P. (2014). Approaches and challenges to optimizing primary care teams’ electronic health record usage. Inform. Prim. Care.

[41] Moon M.C., Hills R., Meniris G. (2018). Understanding optimisation process of electronic health records (EHRs) in select leading hospitals: A qualitative study. BMJ Health Care Inform..

