# Supplementary material for: Pharmacists’ Perspectives on the Use of My Health Record
Source: Pharmacy (Basel). 2020 Oct 14;8(4):190. doi: 10.3390/pharmacy8040190 (PMC7712990; doi:10.3390/pharmacy8040190)
Supplement: Supplementary file 1 [file pharmacy-08-00190-s001.pdf]

Survey questionnaire

What is your gender?

- ○ Male
- ○ Female
- ○ Other

What is your age?

- ○ under 25
- ○ 25–34
- ○ 35–44
- ○ 45–54
- ○ 55–64
- ○ 65+
- ○ Prefer not to answer

Which State/Territory do you practice in?

- ○ ACT
- ○ NSW
- ○ NT
- ○ QLD
- ○ SA
- ○ TAS
- ○ WA
- ○ VIC

Do you practice in a rural or metropolitan area?

- ○ Metropolitan
- ○ Rural

Over the last month, on average, what proportion (in %) have you worked in the following areas of pharmacy? (if you are on leave, what areas have you worked in, over the month prior to the start of your leave)?

Community Pharmacy: _______
Hospital Pharmacy: _______
GP Clinic: _______
Conducting HMR or RMMR: _______
Industrial Pharmacist: _______
Academia/Research: _______
Other: _______

Total: ________

Which of the following is your primary role in which you are engaged in patient care? ***Select only one choice and answer the rest of the questions in this survey based on this role***

- ○ Community Pharmacy
- ○ Hospital Pharmacy
- ○ GP Clinic
- ○ Conducting HMR or RMMR

Do you/ your organization have access to My Health Record?

- ○ Yes
- ○ No
- ○ Unsure

How many times have you accessed My Health Record in the past month?

- ○ 0
- ○ 1–5 times
- ○ 6–10 times
- ○ 11–15 times
- ○ 16 times or more

How likely are you to use My Health Record in the next 12 months?

- ○ Extremely likely
- ○ Somewhat likely
- ○ Neither likely nor unlikely
- ○ Somewhat unlikely
- ○ Extremely unlikely

How much do you agree with the following statements?

	**Strongly Agree**	**Somewhat Agree**	**Neither Agree Nor Disagree**	**Somewhat Disagree**	**Strongly Disagree**
My Health Record will help with continuity of care	○	○	○	○	○
My Health Record reduces medication errors when dispensing	○	○	○	○	○
My Health Record improves the safety of care I provide	○	○	○	○	○
My Health Record improves the quality of patient care I provide	○	○	○	○	○
My Health Record improves my professional relationship with my patients	○	○	○	○	○
My Health Record improves my professional relationship with GPs	○	○	○	○	○

How likely are the following to be **barriers** for you to use My Health Record?

	**Extremely Likely**	**Somewhat Likely**	**Neither Likely Nor Unlikely**	**Somewhat Unlikely**	**Extremely Unlikely**
I am concerned about privacy/ confidentiality issues of My Health Record	○	○	○	○	○
My patients are concerned about privacy/ confidentiality issues of My Health Record	○	○	○	○	○
My workplace does not promote the use of this system	○	○	○	○	○
I do not have the appropriate IT support	○	○	○	○	○
I have not received training to set up the access to the system	○	○	○	○	○
I have not received training to confidently use the system	○	○	○	○	○
It interferes with the existing dispensing processes	○	○	○	○	○

I do not believe the time spent provides a significant value gained	○	○	○	○	○
It is not within my position description in my current professional role	○	○	○	○	○
Other- Please Specify	○	○	○	○	○

How much do you agree with the following statements?

	**Strongly Agree**	**Somewhat Agree**	**Neither Agree Nor Disagree**	**Somewhat Disagree**	**Strongly Disagree**
I have concerns about the security of information within the My Health Record system	○	○	○	○	○
I have concerns about the accuracy of information within the My Health Record system	○	○	○	○	○
My Health Record slows me down when dispensing	○	○	○	○	○
It is not user-friendly to navigate My Health Record to find the information I want	○	○	○	○	○

Overall, I am satisfied with My Health Record

○ Extremely satisfied

○ Somewhat satisfied

○ Neither satisfied nor dissatisfied

○ Somewhat dissatisfied

○ Extremely dissatisfied

What are 3 characteristics (if any) of My Health Record that need changes/improvement?

____

____

____

____

____
